# Neoadjuvant Chemotherapy for Retroperitoneal Sarcoma: A Systematic Review and Meta‐Analysis

**DOI:** 10.1002/jso.70037

**Published:** 2025-07-10

**Authors:** Bernardo Fontel Pompeu, Luiza Soares Guerra, Julia Hoici Brunini, Gabriel Leal Barone, Lucas Monteiro Delgado, Maria Letícia Gobo Silva, Fernando Augusto Batista Campos, Samuel Aguiar Junior

**Affiliations:** ^1^ Department of Surgical Oncology Heliopolis Hospital São Paulo Brazil; ^2^ University of São Caetano do Sul São Paulo Brazil; ^3^ Federal University of Minas Gerais Belo Horizonte Brazil; ^4^ Sarcoma and Bone Tumors Reference Center ‐ A.C. Camargo Cancer Center São Paulo Brazil

**Keywords:** neoadjuvant chemotherapy, retroperitoneal sarcoma, surgical resection, survival outcomes

## Abstract

**Introduction:**

Retroperitoneal sarcoma is a rare malignancy, and surgical resection remains the primary treatment option. While neoadjuvant radiotherapy has shown limited long‐term benefits, the role of neoadjuvant chemotherapy in this setting remains uncertain. This study aimed to evaluate the impact of neoadjuvant chemotherapy on survival outcomes in patients with resectable retroperitoneal sarcoma.

**Methods:**

A comprehensive search was performed in PubMed, Scopus, the Central Register of Clinical Trials, and Web of Science for studies published up to December 2024. Hazard ratios (HRs) with 95% confidence intervals (CIs) were pooled using a random‐effects model, and heterogeneity was assessed using *I*² statistics. Statistical analyses were conducted using R Software version 4.4.1 (R Foundation for Statistical Computing).

**Results:**

Four retrospective studies were included, comprising a total of 2156 patients with resectable retroperitoneal sarcoma, of whom 361 (16.7%) received neoadjuvant chemotherapy. The analysis showed no significant difference in 5‐year overall survival between patients who underwent neoadjuvant chemotherapy and those who did not. A sensitivity analysis, performed after excluding the study contributing most to heterogeneity, revealed a statistically significant 18% higher risk of mortality in patients receiving neoadjuvant chemotherapy (HR 1.18; 95% CI 1.06–1.32). Heterogeneity dropped to *I*² = 0% in this analysis.

**Conclusion:**

These findings suggest that neoadjuvant chemotherapy may be associated with worse survival outcomes, although these results remain exploratory due to the retrospective nature of the included studies and the limited number of available datasets. Ongoing prospective trials, such as the STRASS2 trial, will be critical to further defining the role of neoadjuvant chemotherapy in retroperitoneal sarcoma management.

## 1 Introduction

Retroperitoneal sarcomas (RPS) are rare, comprising 10%–20% of all sarcomas, with an incidence of 0.3%–0.4% per 100 000 population [1, 2, 3, 4, 5, 6, 7, 8]. These tumors typically affect individuals in their fifth decade of life, although they can occur at any age, and are associated with a poor prognosis, with 5‐ and 10‐year survival rates of 51% and 36%, respectively [1, 9, 10, 11]. Surgical resection remains the cornerstone of treatment for localized disease, as chemotherapy and radiotherapy, whether used alone or in combination, have shown no significant benefit without surgery. However, the role of neoadjuvant chemotherapy remains uncertain and highly debated [9, 12, 13, 14].

The STRASS trial did not demonstrate a significant improvement in Abdominal recurrence‐free survival (ARFS) for the overall cohort receiving neoadjuvant radiotherapy compared to surgery alone. However, subgroup analysis suggested a potential benefit in patients with liposarcomas, where radiotherapy appeared to delay local recurrence. These findings highlighted the importance of histologic subtype in treatment response and reinforced the need for personalized treatment approaches in RPS. Although the STRASS trial focused on radiotherapy, its results underscore the complexity of managing RPS and the ongoing debate around the benefit of neoadjuvant strategies, providing a relevant context for evaluating the impact of neoadjuvant chemotherapy in this setting [14]. Similarly, the STREXIT study reinforced that high‐risk tumors, such as Grade 3 (dedifferentiated liposarcomas) DDLPS and (leiomyosarcomas) LMS, are less responsive to locoregional treatments and may benefit more from systemic therapy [15]. Another large retrospective study showed that Grade 3 DDLPS and LMS are more likely to develop distant recurrences compared to other subtypes [16]. Based on these findings, the ongoing STRASS II trial attempts to evaluate the impact of neoadjuvant chemotherapy followed by surgery compared to surgery alone on disease‐free survival (DFS) [17].

Limited studies have directly evaluated the outcomes of perioperative chemotherapy in retroperitoneal sarcomas [3, 9, 18, 19]. Most analyses rely on national database data to explore the impact of this treatment modality on improving outcomes [4, 5, 6]. Moreover, no meta‐analyses have been conducted, and existing reviews primarily assess the effects of chemotherapy across all modalities, whether neoadjuvant, adjuvant, or combined with radiotherapy [20]. While awaiting the results of the STRASS II trial, we conducted a meta‐analysis to evaluate the impact of neoadjuvant chemotherapy on overall survival in patients with retroperitoneal sarcoma.

## 2 Materials and Methods

This systematic review followed the Preferred Reporting Items for Systematic Reviews and Meta‐Analysis (PRISMA) guidelines [21]. The study protocol was registered in the International Prospective Register of Systematic Reviews (PROSPERO) with registration number CRD42024628743 [22].

### 2.1 Search Strategy

A systematic search was performed on PubMed, Cochrane Central Register of Clinical Trials, Web of Science, and Scopus for studies published up to December 2024. The search strategy was as follows: (“Retroperitoneal sarcoma” OR “sarcoma of the retroperitoneum”) AND (“perioperative chemotherapy” OR “neoadjuvant chemotherapy” OR “preoperative chemotherapy” OR “surgery alone” OR “surgical resection” OR “surgery only”).

### 2.2 Eligibility Criteria

We included studies comparing neoadjuvant chemotherapy versus no neoadjuvant chemotherapy in patients with resectable retroperitoneal sarcomas. The exclusion criteria were: (1) limb or trunk soft tissue sarcoma therapies, (2) studies lacking a control group, (3) publications that were not suitable for inclusion, such as case reports, conference abstracts, meta‐analyses, reviews, and animal experiments; (4) overlapping populations. However, concerning overlap studies, if different periods or outcomes were addressed in the reports, both papers were considered for the analysis.

### 2.3 Data Extraction and Endpoints

Two authors (B.F.P. and L.S.G.) independently screened the articles for inclusion criteria and extracted data from the selected studies. Any disagreements were resolved by consensus or, if necessary, by consulting a third author (S.A.J.). The primary outcome assessed was a 5‐year OS.

### 2.4 Quality Assessment

The evaluation of non‐randomized studies was carried out using the Cochrane Collaboration tool for assessing the risk of bias in non‐randomized studies (ROBINS‐I) [23]. In this assessment, each study was categorized as critical, serious, moderate, or low risk in the seven domains: confounding, selection, classification, deviations from intended interventions, missing data, measurement of outcomes, and selection of reported results. Two authors (B.F.P and L.S.G) independently assessed the risk of bias, and consensus resolved disagreements. Publication bias was not assessed through visual inspection of funnel plot asymmetry due to the inclusion of fewer than 10 studies, which is considered insufficient for reliable evaluation.

### 2.5 Statistical Analysis

We pooled hazard ratios (HRs) with 95% confidence intervals (CIs) using a random‐effects model. Statistical significance was defined as *p* < 0.05. Heterogeneity was assessed using the Cochran *Q* test and the *I*² statistic. *I*² values were interpreted as follows: less than 25% indicated low heterogeneity, 25% to 50% indicated moderate heterogeneity, and greater than 50% indicated high heterogeneity [24]. For outcomes with substantial heterogeneity, we used Baujat plots to evaluate each study's contribution to the overall effect size and heterogeneity. Additionally, leave‐one‐out sensitivity analyses were conducted by systematically removing individual studies to assess the robustness of the pooled estimates. All statistical analyses were performed using R Software (version 4.4.1, R Foundation for Statistical Computing).

## 3 Results

### 3.1 Study Selection and Characteristics

**Figure 1:**
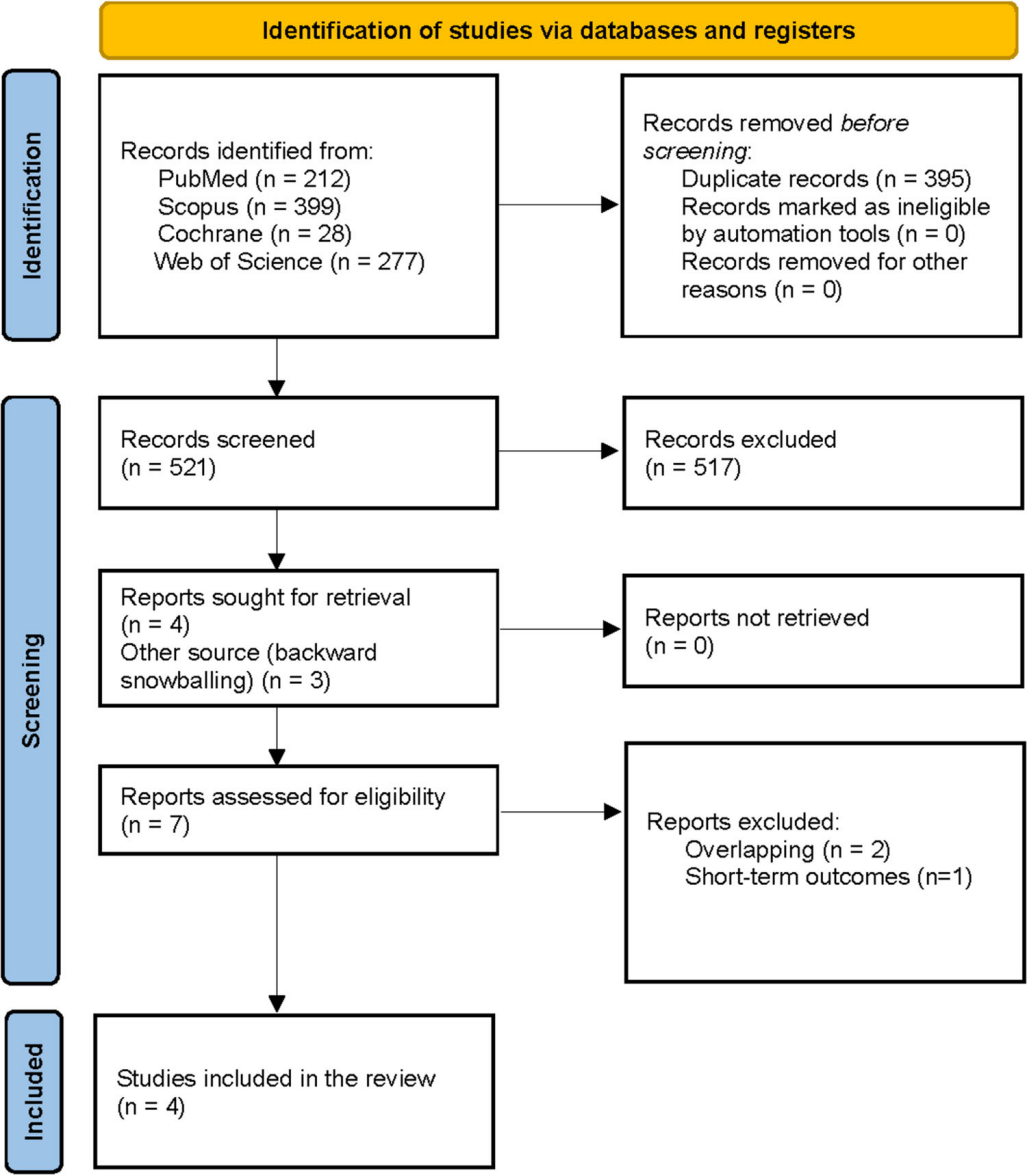
PRISMA flow diagram of study screening and selection.

As shown in Figure 1, the initial database search yielded 916 records. After removing 395 duplicates and excluding 517 records based on titles and abstracts, three additional studies were identified through backward snowballing. In total, four retrospective observational studies were included in the final analysis [3, 4, 5, 9].

The studies involved 2156 patients with resectable retroperitoneal sarcoma, all of whom underwent surgical resection [3, 4, 5, 9]. Among them, 361 patients (16.7%) received neoadjuvant chemotherapy before surgery, while the remaining 1795 patients (83.3%) underwent upfront surgery, either alone or in combination with adjuvant therapies such as chemotherapy or radiotherapy [3, 4, 5, 9]. Females comprised 52.7% of the cohort. The mean age at diagnosis was 54.38 ± 11.71 years. Charlson scores were grouped according to increasing comorbidity burden, where 0 indicates no comorbidities, and higher scores reflect progressively greater systemic disease severity. The overall distribution was as follows: 59.4% for score 0, 8.3% for score 1, 1.5% for score 2, 0.1% for score 3, and 30.7% for unknown scores. Tumor histology includes sarcomas not otherwise specified (28.1%), liposarcoma (25.7%), leiomyosarcoma (24.5%), undifferentiated pleomorphic sarcoma (14.4%), fibrosarcoma (1.6%), malignant peripheral nerve sheath tumors (1.7%), and other types (4%). Tumor grade was distributed as follows: Low (19.88%), intermediate (18.15%), and high (61.98%). Additionally, the average size of the tumor was 21.3 ± 10.76 cm. The surgical margin distribution showed R0 margins in 41.03% of cases, R2 margins in 19.87%, and R1 margins in 13.51%. Additionally, 25.60% of cases were classified as unknown. The mean follow‐up period was 46.9 ± 38.2 months.

Two studies utilized the NCDB database, covering distinct periods, thereby minimizing the risk of cohort overlap [4, 5]. To further avoid duplication, we evaluated all eligible studies for potential overlap. Specifically, the study by Ma et al. (2020) [6], although relevant, was excluded due to potential overlap with the cohorts analyzed in Miura et al. (2015) and Tortorello et al. (2023) [4, 5], both of which also utilized NCDB data [4, 5]. The exclusion was justified by overlapping time frames, shared database origin, and similar cohort definitions. We prioritized the most recent and comprehensive datasets to ensure robustness of the analysis.

Patients were classified under the neoadjuvant chemotherapy (NCH) group if they received chemotherapy before surgery, either alone or in combination with other modalities [3, 4, 5, 9]. When studies provided detailed treatment timing, we extracted data related explicitly to preoperative chemotherapy [3, 4, 5, 9]. Bremjit et al. and Tortorello et al. reported NCH rates of 21.2% and 6.3%, respectively [3, 5]. Miura et al. described 10.6% of patients receiving NCH [4]. In Singer et al. 17% of patients were treated preoperatively, although some also received postoperative chemotherapy [9]. When timing or regimen separation was unclear, patients were included under the NCH group only when chemotherapy was explicitly administered before surgery. Study characteristics are detailed in Tables 1 and 2.

**Table 1:** Baseline characteristics of observational studies included.

Author (Year)	Country	Ch/No‐Ch	Total	Design	Sex, Female, *n* (%) Ch/No‐Ch	Age at diagnosis, years Ch/No‐Ch(Mean ± SD)	Charlson Comorbidity Score, *n* (%)	Study period
Bremijit 2014	USA	28/57	85	R‐Obs	77 (58.3)	55 ± 12	NA	2000 to 2013
Miura 2015	USA	175/1525^a^	3050	R‐Obs (PSM)	801 (52.5)/814 (53.4)	53.5 ± 6.0/56 ± 4.62	0: 856 (56.1)/898 (58.9) 1: 116 (7.6)/112 (7.3) 2: 25 (1.7)/18 (1.2) Unknown:528 (34.6)/497 (32.6)	1998 to 2011
Tortorello 2023	USA	144/144	288	R‐Obs (PSM)	76 (52.8)/76 (52.8)	59.5 ± 3.1/60 ± 4.04	0: 115 (79.9)/113 (78.5) 1: 24 (16.7)/26 (18.1) 2: 3 (2.1)/3 (2.1) 3: 2 (1.4)/2 (1.4)	2014 to 2019
Singer 1995	USA	14/69	83	R‐Obs (PSM)	39 (47)	48 ± 19.05	NA	1975 to 1994

*Note:* Charlson Comorbidity: Class 0 (no comorbidities), Class 1 (light comorbidities), Class 2 (moderate comorbidities), Class 3 (severe comorbidities), and Class 4 or more (multiple or very severe comorbidities).

Abbreviations: Ch, neoadjuvant chemotherapy; N/A, not available; PSM, propensity score matching; R‐obs, retrospective observational study.

a 175 patients received Neoadjuvant Chemotherapy.

**Table 2:** Surgical characteristics of the studies included in the meta‐analysis.

Author (Year)	Ch/No‐Ch	Histology	Tumor grade, *n* (%), Ch/No‐Ch	Margin status, *n* (%), Ch/No‐Ch	Tumor size, cm Ch/No‐Ch	Reported chemotherapy/radiotherapy use	Surgery
Bremijit 2014	28/57	LMS—29 (22.0) LPS—80 (60.6) Sarcoma (Other)—23 (17.4)	Low 48 (38.4) Intermediate 43 (34.4) High 34 (27.2)	R0—60 (47.6) R1—59 (46.8) R2—7 (5.6)	29 ± 15.01	Chemotherapy: 21.2% neoadjuvant. RT: 30.3% neoadjuvant.	Number of. Organs resected: 0: 30 (24.2) 1: 45 (36.3) ≥ 2: 49 (39.5)
Miura 2015	1525/1525	LPS: 312 (20.4)/317 (20.8) LMS: 381 (25)/418 (27.4) UP: 263 (17.2)/266 (17.4) Fibro: 29 (1.9)/29 (1.9) MPNST: 25 (1.7)/20 (1.3) b Unsp: 515 (33.8)/475 (31.2)	Low: 110 (7.2)/120 (7.9) Moderate: 146 (9.6)/122 (8) High: 460 (30.2)/455 (29.8) a Un: 809 (53)/828 (54.3)	R0: 614 (40.3)/629 (41.2) R1: 178 (11.7)/192 (12.6) R2: 315 (20.6)/309 (20.3) a Un: 418 (27.4)/395 (25.9)	< 10: 409 (26.8)/423 (27.7) 10–20: 526 (34.5)/534 (35) > 20: 270 (17.7)/286 (18.8) a Un: 320 (21)/282 (18.5)	Chemotherapy: 10.6% neoadjuvant. ** RT: 10.8% overall.	Limited: 515 (33.8)/544 (35.7) c Rd/Dbk: 887 (58.2)/873 (57.2) Surgery: 123 (8)/108 (7.1)
Tortorello 2021	144/144	DLPS: 67 (46.5)/68 (47.2) LMS: 77 (53.5)/76 (52.8)	2: 27 (18.8)/26 (18.1) 3: 77 (53.5)/77 (53.5) 4: 40 (27.8)/41 (28.5) #	NA	17.5 ± 3.75/17.4 ± 4.39	Chemotherapy: 6.3% neoadjuvant. ** RT: 32.3% overall.	NA
Singer 1995	14/69	LMS: 39 (47). LPS: 12 (14.5) MPNST: 7 (8%) Others: 25 (30.5)	Low: 25 (30) Intermediate: 14 (17) High: 44 (53)	NA	< 5: 7 (8.5) 5–10 cm: 35 (42) > 10 cm: 41 (49.5)	Chemotherapy: 17% preoperative. RT: 2% preoperative, 29% postoperative.	NA

Abbreviations: Ch, neoadjuvant chemotherapy; DLPS, dedifferentiated liposarcoma; Fibro, fibrosarcoma; LMS, leiomyosarcoma; LPS, liposarcoma; MPNST, malignant peripheral nerve sheath tumor; RT, radiotherapy; UP, undifferentiated pleomorphic.

** Radiotherapy Timing not reported.

^a^ Unknown.

^b^ Unspecified.

^c^ Rd/Dbk: Radical/Debulking. #: For consistency, tumor grading in Tortorello et al. was harmonized as low/intermediate (Grades 1–2) and high (Grades 3–4), due to the absence of FNCLCC or CINSARC classification in the NCDB.

### 3.2 Pooled Analyses of All Studies

#### 3.2.1 Overall Survival and Sensitivity Analyses

**Figure 2:**
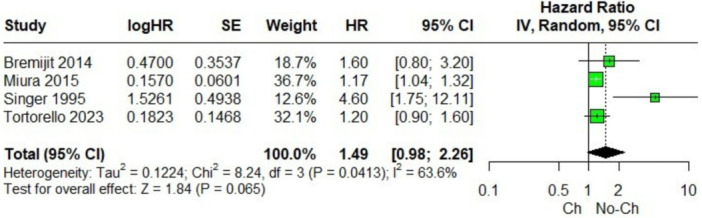
Forest plots of 5‐year OS, the hazard ratio for effect size data. Comparison between neoadjuvant chemotherapy vs. no chemotherapy.

**Figure 3:**
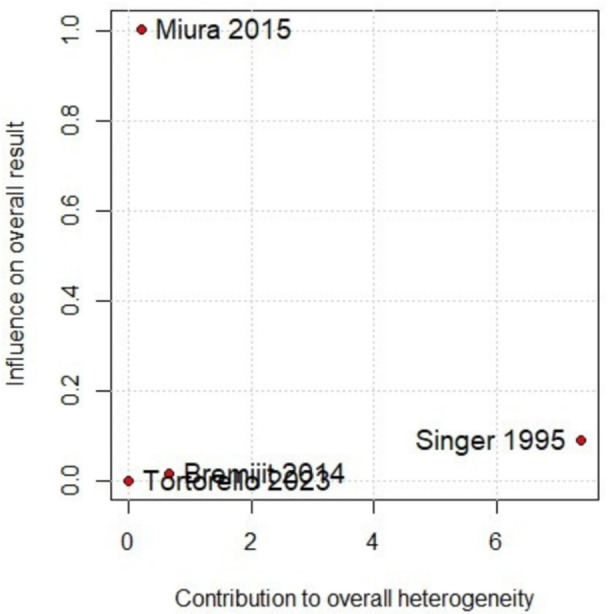
Baujat plot of 5‐year OS.

**Figure 4:**
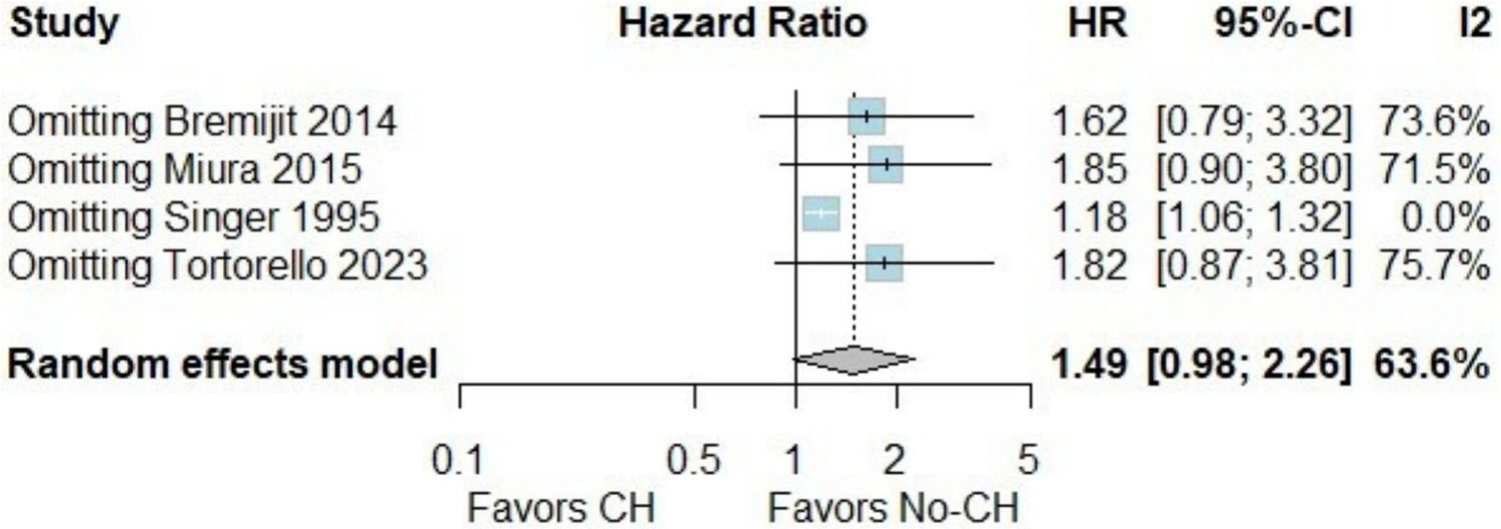
Leave‐one‐out plot of 5‐year OS.

**Figure 5:**
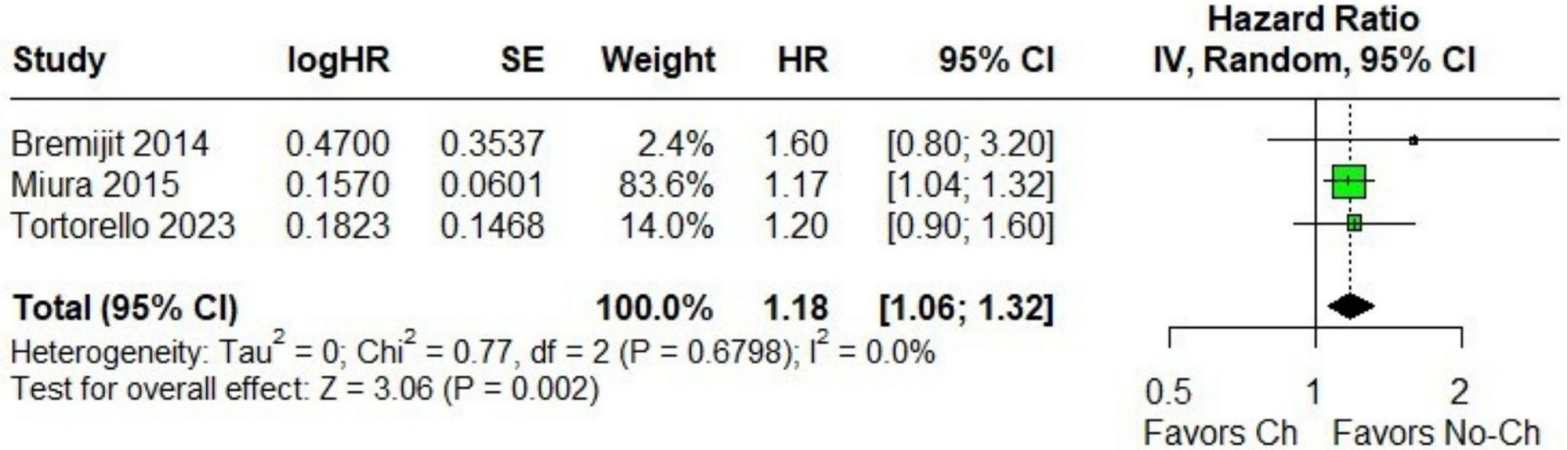
Forest plots of 5‐year OS, the hazard ratio for effect size data. Comparison between neoadjuvant chemotherapy vs. no chemotherapy (excluding Singer 1995).

In the pooled analysis of patients with retroperitoneal sarcoma who underwent or did not undergo neoadjuvant chemotherapy, no significant differences were observed in 5‐year OS (Figure 2) [3, 4, 5, 9]. However, there was considerable heterogeneity among the included studies. The Baujat plot analysis revealed that one study contributed substantially to this heterogeneity. Specifically, the study by Singer et al. was identified as the primary contributor to the variability in the outcomes (Figure 3) [9]. When this study was excluded in a leave‐one‐out sensitivity analysis, the heterogeneity was significantly reduced, with the *I*² dropping to 0%. This adjustment provided a clearer interpretation of the pooled results, as shown in Figures 4 and 5. Excluding Singer et al. the adjusted hazard ratio (HR) for 5‐year OS was 1.18 (95% CI: 1.06–1.32) [9], indicating a statistically significant 18% higher risk of mortality for patients who underwent neoadjuvant chemotherapy compared to those who did not.

### 3.3 Quality Assessment

**Figure 6:**
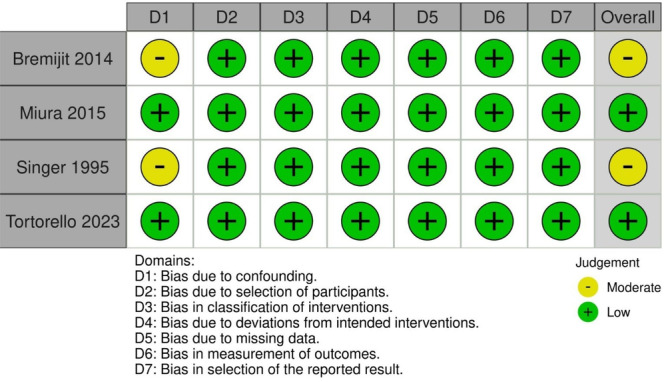
Critical appraisal of studies according to the Cochrane Collaboration's tool for assessing risk of bias—ROBINS‐I.

The individual assessment of each study included in the meta‐analysis is presented in Figure 6 [3, 4, 5, 9]. Among the four studies evaluated, two were categorized as having a moderate risk of bias (Bremijit 2014 and Singer 1995) due to confounding, while the remaining two were classified as having a low risk of bias (Miura 2015 and Tortorello 2023) [3, 4, 5, 9].

## 4 Discussion

In this systematic review and meta‐analysis, we evaluated the impact of neoadjuvant chemotherapy on overall survival in patients with retroperitoneal sarcoma across four observational studies. No significant differences in 5‐year survival were observed in the initial analysis [3, 4, 5, 9]. However, considerable heterogeneity was identified, primarily driven by the study by Singer et al. [9]. After excluding this study, the analysis revealed a higher risk of mortality for patients who received neoadjuvant chemotherapy.

Surgery is the primary treatment for retroperitoneal sarcomas, despite the anatomical complexity and the frequent need for multivisceral resections to achieve R0 margins [9, 12, 13, 14, 25]. Attempts to combine surgery with other treatment modalities to improve outcomes have largely failed, resulting in high recurrence rates and poor prognosis [3, 9, 18, 19]. The greater the surgical complexity, the more difficult it becomes for patients to tolerate chemotherapy regimens, resulting in the loss of the oncological benefits of adjuvant treatments [5].

The rationale for neoadjuvant therapy is enabling patients to complete all cycles effectively, theoretically achieving a greater oncological impact [5, 18, 26]. This approach aims to address micrometastases, promote tumor response, sterilize the tumor capsule, and provide a better understanding of the tumor's biological behavior [5, 18, 26]. However, the response to neoadjuvant systemic therapy in high‐risk retroperitoneal sarcoma has been modest. Tseng et al. conducted a multi‐institutional study analyzing 158 patients with primary, high‐risk retroperitoneal sarcomas treated with neoadjuvant chemotherapy [26]. They reported a 23% partial response rate, 56% with stable disease, and 21% experiencing disease progression, which was significantly associated with poorer overall survival [26]. Their findings underscore the challenges in achieving meaningful tumor regression and suggest that disease progression during therapy may serve as a prognostic indicator for surgical outcomes [26]. Further, the failure of radiotherapy and chemotherapy as adjuvants in the treatment of retroperitoneal sarcomas may be linked to the fact that most cases involve large tumors with a high risk of systemic metastases, where local therapies have limited effectiveness [14]. The STRASS trial, while demonstrating no overall benefit of preoperative radiotherapy, highlighted potential advantages for specific histological subtypes, such as low‐grade retroperitoneal sarcomas and liposarcomas, whereas leiomyosarcomas and high‐grade tumors did not show similar benefits [17].

Our analysis did not initially demonstrate statistical significance when comparing neoadjuvant chemotherapy to non‐chemotherapy. To explore the impact of heterogeneity, we conducted a sensitivity analysis excluding the Singer cohort, which resulted in a notable change in the pooled outcome. The Singer study, which analyzed 83 patients treated for retroperitoneal sarcoma from 1975 to 1994, was the primary source of heterogeneity in our analysis due to its large hazard ratio (HR 4.60; 95% CI: 1.75–12.11; *p* = 0.002) and significant weight on the Baujat plot [9]. Importantly, this study reflects a period with notable differences in clinical practice. Since then, advancements in the management of critically ill patients have occurred, including improvements in medication, chemotherapy protocols, radiology, surgical techniques, anesthetics, and access to intensive care. These advancements may account for the differences in outcomes observed over time. After its exclusion, we observed an increase in mortality of up to 18% in the group undergoing neoadjuvant chemotherapy.

It is noteworthy that our cohort consisted primarily of high‐grade and high‐risk tumors for systemic metastases, including dedifferentiated liposarcoma, leiomyosarcoma, and undifferentiated pleomorphic sarcoma. Furthermore, chemotherapy is accompanied by certain disadvantages, such as potentially prolonging the time to surgery or elevating the risk of surgical complications due to patient deconditioning, which ultimately reduces the ability to recover from a major operation [5, 18]. Additionally, the administration of neoadjuvant chemotherapy may delay the initiation of radiotherapy. These factors could explain the poorer prognosis observed in these patients, further increasing their mortality risk. In agreement with our findings, Ma et al. conducted a retrospective analysis using the NCDB database to evaluate patients undergoing different treatment modalities for retroperitoneal sarcoma [6]. The study highlighted that neoadjuvant chemotherapy was associated with lower overall survival compared to surgery alone, with a hazard ratio of 1.54 (95% CI: 1.27–1.88; *p* < 0.001) [6]. Unfortunately, we excluded this study from our analysis due to overlapping samples with the Tortorello et al. and Miura et al. works, which could have compromised the validity and reliability of our results [4, 5]. In another study, Schwartz et al. analyzed 571 patients with retroperitoneal sarcomas, with liposarcoma (34%) and leiomyosarcoma (28%) being the most common subtypes [27]. Median disease‐free survival (DFS) was 35.3 months, and overall survival (OS) was 81.6 months [27]. Poorer outcomes were linked to high‐grade tumors, nodal‐positive disease, multifocality, and incomplete resections (R2) [27]. The study highlighted that tumor‐specific characteristics strongly predict survival, while complete surgical resection (R0/R1) remains the most critical modifiable factor, with nonsurgical therapies showing no significant benefit [27]. Finally, Perez et al. analyzed 312 patients with truncal and retroperitoneal sarcomas, identifying liposarcoma (35.9%) and leiomyosarcoma (30.1%) as the most common subtypes [19]. Median overall survival was 74 months, with worse outcomes linked to higher tumor grade and multifocal disease [19]. While initial analysis suggested a survival benefit from neoadjuvant chemotherapy, multivariate regression did not confirm it as an independent predictor [19].

The higher prevalence of liposarcomas, leiomyosarcomas, and high‐grade tumors plays a significant role in prognosis [8, 16, 19, 25, 27]. A study conducted by the French Sarcoma Group highlighted the importance of histology as an independent determinant of clinical outcomes [28]. In a retrospective analysis of 586 patients, the researchers identified that characteristics such as tumor grade and involvement of adjacent organs varied according to histological subtype and were directly associated with local recurrence, abdominal sarcomatosis, and distant metastases [28]. The dedifferentiated liposarcomas, Grade 3, were identified as an independent prognostic factor for poorer overall survival [28].

This study has several limitations. First, the observational and retrospective design of the included studies precludes causal inference and may introduce inherent biases. Second, considerable heterogeneity in study design, treatment protocols, and patient populations complicates the interpretation of pooled outcomes, necessitating sensitivity analyses to explore variability. Third, some studies were based on large national databases, which, while comprehensive, may suffer from incomplete data and limited clinical detail. Fourth, in certain studies, chemotherapy was broadly categorized, and neoadjuvant chemotherapy was analyzed only as a subgroup, potentially diluting its specific effect. Fifth, the number of patients receiving neoadjuvant chemotherapy was relatively small compared to the control group, limiting the statistical power and generalizability of results. Sixth, only four studies met the eligibility criteria, which restricts the evidence base and limits the ability to perform subgroup analyses or assess publication bias. Seventh, the inclusion of older studies with outdated clinical practices may distort pooled estimates when combined with more recent data. Finally, the analysis was not based on individual patient data, which prevented stratification by tumor grade, a key prognostic factor in retroperitoneal sarcomas. These limitations highlight the need for well‐designed, prospective randomized trials, such as the ongoing STRASS2 study, to better define the role of neoadjuvant chemotherapy in this setting.

## 5 Conclusion

This systematic review and meta‐analysis, including 2156 patients with resectable retroperitoneal sarcoma, found no significant difference in 5‐year overall survival between patients who received neoadjuvant chemotherapy and those who did not. Notably, only 361 patients (16.7%) in the pooled cohort underwent neoadjuvant chemotherapy, which limits the statistical power of the comparison. A sensitivity analysis, performed after excluding the study that contributed most to heterogeneity, suggested an 18% higher risk of mortality associated with neoadjuvant chemotherapy. These findings remain exploratory due to the retrospective nature of the included studies, and the ongoing STRASS2 trial is expected to provide more definitive evidence regarding the role of neoadjuvant chemotherapy in the management of retroperitoneal sarcomas.

## Author Contributions

Conception and design – Bernardo Fontel Pompeu, Samuel Aguiar Junior, Maria Letícia Gobo Silva, Fernando Augusto Batista Campos. Data acquisition – Bernardo Fontel Pompeu, Luiza Soares Guerra, Julia Hoici Brunini, Lucas Monteiro Delgado, Gabriel Leal Barone. Data analysis – Bernardo Fontel Pompeu, Luiza Soares Guerra, Julia Hoici Brunini, Lucas Monteiro Delgado, Gabriel Leal Barone. Data interpretation – Bernardo Fontel Pompeu, Luiza Soares Guerra, Julia Hoici Brunini, Lucas Monteiro Delgado, Gabriel Leal Barone, Samuel Aguiar Junior, Maria Letícia Gobo Silva, Fernando Augusto Batista Campos. Drafting and editing the manuscript – Bernardo Fontel Pompeu, Luiza Soares Guerra, Julia Hoici Brunini, Lucas Monteiro Delgado, Gabriel Leal Barone, Samuel Aguiar Junior, Maria Letícia Gobo Silva, Fernando Augusto Batista Campos. Revising it critically for important intellectual content – Bernardo Fontel Pompeu, Samuel Aguiar Junior, Maria Letícia Gobo Silva, Fernando Augusto Batista Campos. All authors approved the final version of the article, including the authorship list.

## Disclosure

The authors have nothing to report.

## Synopsis

This meta‐analysis investigates the effect of neoadjuvant chemotherapy on survival in resectable retroperitoneal sarcoma and finds no significant benefit; sensitivity analysis suggests possible harm.

## Data Availability

The authors have nothing to report.
